# The impact of glucose tolerance state on seropositivity rate after hepatitis B vaccination

**DOI:** 10.1038/s41598-022-07163-x

**Published:** 2022-02-23

**Authors:** Maria Mercedes Chang Villacreses, Rudruidee Karnchanasorn, Horng-Yih Ou, Raynald Samoa, Lee-Ming Chuang, Ken C. Chiu

**Affiliations:** 1 Department of Clinical Diabetes, Endocrinology, and Metabolism, City of Hope National Medical Center, Duarte, CA USA; 2 Division of Endocrinology, Metabolism and Nutrition, Department of Internal Medicine, Harbor-UCLA Medical Center, Torrance, CA USA; 3 Division of Endocrinology, Department of Medicine, University of Kansas Medical Center, Kansas City, KS 66160 USA; 4 Division of Endocrinology and Metabolism, Department of Internal Medicine, National Cheng-Kung University Medical College and Hospital, Tainan, Taiwan; 5 Department of Internal Medicine, National Taiwan University Hospital, Taipei, Taiwan; 6 Institute of Clinical Medicine, College of Medicine, National Taiwan University, Taipei, Taiwan; 7 The Lundquest Institute, Torrance, CA USA

**Keywords:** Immunology, Endocrinology

## Abstract

Immunization is recommended for people with diabetes mellitus (DM), but little information is available on their seropositivity rates. To determine the impact of glucose tolerance state on seropositivity rate after hepatitis B vaccination, we included 7645 adult participants from the National Health and Nutrition Examination Survey 2005–2016 who reported three doses of hepatitis B vaccine and were seropositive for anti-hepatitis B surface antibody (≥ 12.0 mIU/mL), after exclusion of those positive for anti-hepatitis B core antibody and/or hepatitis B surface antigen. We classified the states of glucose tolerance as normal glucose tolerance (NGT, 61.68%), abnormal glucose tolerance (AGT, 26.02%), or DM (13.30%). We observed a stepwise decline in hepatitis B seropositivity rate from NGT (53.64%) to AGT (45.52%) to DM (28.84%) (P < 0.0001). We confirmed these results after standardization for age and BMI (P < 0.0001 for all subgroup analyses) and in subgroup analyses by gender and racial/ethnic group. Dysregulated glucose metabolism is associated with a decreased seropositivity rate after hepatitis B vaccination. Our observations suggest that regular follow-up screening for anti-hepatitis B surface antibody, with additional booster vaccination as necessary, is especially important in patients with DM. Whether a similar phenomenon exits for other vaccines, especially COVID-19, remains to be investigated.

## Introduction

The United States Centers for Disease Control and Prevention (CDC) recommends that adults with diabetes mellitus (DM) receive influenza, pneumococcal, Tdap (tetanus toxoid, reduced diphtheria toxoid, and acellular pertussis), hepatitis B, and Zoster vaccines; this is endorsed by the American Diabetes Association (ADA) as a standard of medical care in diabetes^1^. These recommendations were based on various observations. For example, exacerbated hyperglycemia is frequently observed during infection in patients with DM^2^. In particular, an outbreak of hepatitis B infection in patients with DM was reported from reusable lancet devices^3^. DM increases the risk of various infections and associated infection-related mortality^4^, which could potentially be reduced through proper vaccination. Thus, immunization of people with DM offers the best protection against vaccine-preventable diseases.

Although vaccination is highly recommended in people with DM, there is limited information available regarding serological responses after vaccination among this population. Most results to date come from studies of influenza vaccination, but offer conflicting information. Among older adults, those with DM demonstrated a significantly lower immunogenicity profile in response to the influenza vaccine than those without DM^5^. In contrast, some reports revealed no difference in the immunogenicity of the influenza vaccine in patients with DM^6,7^. Although the benefit of influenza vaccination was confirmed in a systematic review, the authors also emphasized a lack of high-quality randomized controlled trials of seasonal influenza vaccination in patients with DM and suggested that the observed benefit may arise from residual confounding factors but not the vaccine itself^8^. Furthermore, the presence or increasing titer of antibody after vaccination could be the result of either vaccination itself or concurrent infection.

With multiple serological markers available, hepatitis B presents a unique opportunity to address the issue of the source of seropositivity. We used immunization history and the measurement of anti-hepatitis B surface antibody (anti-HBs), anti-hepatitis B core antibody (anti-HBc), and hepatitis B surface antigen (HBsAg) to determine the impact of glucose tolerance state on seropositivity rate of anti-hepatitis B surface antibody after hepatitis B vaccination in a representative adult population from the United States of America.

## Methods

### Participants

The data used in this study was extracted from the National Health and Nutrition Examination Survey (NHANES) from 2005 to 2016 (https://wwwn.cdc.gov/nchs/nhanes/Default.aspx). The study was approved since 1987 initially by the NHANES Institutional Review Board and then since 2003 by the Research Ethics Review Board of the National Center for Health Statistics (NCHS), CDC under the study Protocol #2005–06 for 2005–2006, Continuation of Protocol #2005–06 for 2007–2008 and 2009–2010, Protocol #2011–17 for 2011–2012, and Continuation of Protocol #2011–17 for 2013–2014 and 2015–2016. All procedures in the NHANES were performed in accordance with relevant guidelines and regulations. Written informed consents were obtained from all participants at entry of survey. Analysis of de-identified data from the survey is exempt from the federal regulations for the protection of human research participants (https://www.cdc.gov/rdc/b6pubeyond/PuB600.htm). The data was de-identified before release and only anonymous data was used in the present study. Adult participants (20 years or older) who self-reported receiving at least three doses of hepatitis B vaccine and had a documented serostatus for hepatitis B following vaccination, a body mass index (BMI) measurement, and a defined glucose tolerance state were included in this study (n = 7645). The information of hepatitis B vaccination was obtained by an interviewer who administered the questionnaires in a standardized fashion on the day of survey. However, the time of vaccination and the serostatus after the completion of hepatitis B vaccination were not included in the survey. The participants who reported to receive three doses of hepatitis B vaccine were included in this study without any prior information of serostatus of hepatitis B before the survey. All subjects who met the enrollment criteria of this study were included regardless immune competence, the risk of hepatitis B infection, and other factors that might affect the immune response. The samples for the determination of hepatitis B serostatus were obtained on the day of survey with the oral glucose tolerance tests.

### Hepatitis B serostatus

This study only included participants who had received at least three doses of hepatitis B vaccine, and who were positive for anti-hepatitis B surface antibody, and negative for both anti-hepatitis B core antibody (indicative of natural infection) and hepatitis B surface antigen (indicative of being a carrier).

### Glucose tolerance state

Participants who were diagnosed with DM by a health care provider, and/or were receiving any anti-diabetic agents, and/or had one of the following glucose measurements were classified as having DM: hemoglobin A1C (A1C) ≥ 48 mmol/mol (6.5%), fasting plasma glucose (FPG) concentration ≥ 7.0 mmol/L (126 mg/dL), and/or 2-h post-challenged plasma glucose (2hPG) concentration ≥ 11.1 mmol/L (200 mg/dL). After separation of participants with DM, those with A1C of 39–47 mmol/mol (5.7–6.4%), FPG concentration of 5.6–6.9 mmol/L (100–125 mg/dL), and/or 2hPG concentration of 7.8–11.0 mmol/L (140–199 mg/dL) were defined as having abnormal glucose tolerance (AGT). The remaining participants with A1C < 39 mmol/mol (5.7%), FPG concentration < 5.6 mmol/L (100 mg/dL), and/or 2hPG concentration < 7.8 mmol/L (140 mg/dL) were defined as having normal glucose tolerance (NGT).

### Laboratory measurements

Plasma glucose concentration was determined in the NHANES study using a hexokinase-based method. Due to a different assay being used in the cycle of NHANES 2005–2006, a regression equation was required to realign plasma glucose concentration from the cycles of NHANES 2005–2006 with other cycles of NHANES, as recommended by NHANES. A1C was determined in the NHANES study using an HPLC analytical column. Because the laboratories were standardized by participating in the National Glycohemoglobin Standardization Program (NGSP), no realignment was required.

The VITROS Anti-HBs, Anti-HBc, and HBsAg assays (Ortho Clinical Diagnostics, Raritan, New Jersey, USA) were used in this study for 2007–2016 with standardized calibration and quality control protocols as described at the NHANES website (https://wwwn.cdc.gov/nchs/nhanes/Default.aspx). The measuring range of Anti-HBs was 4.23–1000 mIU/mL. A result of < 5.00 mIU/mL indicated to be negative while a result of ≥ 12.0 mIU/mL indicated to be positive. For an initial result of > 5.00 mIU/mL and < 12.0 mIU/mL, the sample was retested in duplicate. If both repeats were < 5.00 mIU/mL, the sample was reported as negative. If both repeats were ≥ 12.0 mIU/mL, the sample was reported as positive. The result was indeterminate if one or both replicate results were > 5.00 mIU/mL and < 12.0 mIU/mL. For 2005–2006, Abbott AUSAB Enzyme Immunoassay kit was used for anti-hepatitis B surface antibody with standardized calibration and quality control protocols. The detection limit was 0.050 mIU/mL. A result of < 10 mIU/mL was defined as negative while a result of ≥ 10 mIU/mL was defined as negative. Although different assays were used in NHANES 2005–2006 for the determination of hepatitis B serology, the relevant NHANES quality assurance and quality control protocols met the 1988 Clinical Laboratory Improvement Act mandates, and no realignment was required for the qualitative results. Only the qualitative results were released by the NHANES.

### Assessment

Based on self-report by participants, gender was defined as male or female and race/ethnicity was defined as Mexican American, Other Hispanic, non-Hispanic white, non-Hispanic black, or other. Age in years, at the time of the screening interview, was calculated based on the self-reported date of birth. BMI was calculated as weight in kilograms divided by height in meters squared. Hepatitis B vaccination was based on self-report of at least three doses, less than three doses, or no doses.

### Statistical analyses

SYSTAT 13 (Systat Software, Inc.) was used in this study. Due to a complex sampling scheme with an oversampling strategy, sample-weighted analyses were performed as recommended by NHANES. Sample-weighted results are presented unless otherwise specified. Proportional variables are presented as counts with percent, whereas continuous variables are presented as means with standard deviation. Proportional variables were compared using chi-square tests. Continuous variables were compared using Student’s t-tests or ANOVA tests as appropriate. Test factor standardization using multiway tables statistically removes the effect of control variables so that the relationship between independent and dependent variables can be examined without these control variables. BMI was converted to the nearest integer greater than or equal to the number before standardization. We considered a nominal P value of less than 0.05 as significant.

## Results

### Participant selections and clinical characteristics

Adult participants (20 years or older) who received a hepatitis B vaccine were included in this study to determine the impact of glucose tolerance state on seropositivity rate after hepatitis B vaccination. The NHANES 2005–2016 included 60,936 participants. Among them, 34,180 participants were 20 years or older. Given that BMI is an important determinant of glucose dysregulation, participants without a BMI measurement were excluded (n = 1736). Participants whose glucose tolerance state could not be defined by a history of established diabetes, use of anti-diabetic agents, A1C, FPG, and/or 2hPG concentration were also excluded (n = 1,371). Due to a lower seropositivity rate of anti-hepatitis B surface antibody in participants who received less than three doses of hepatitis B vaccine (n = 787) only those who had received at least three doses (32.91% vs. 50.01%, respectively, P < 0.0001) were included, yielding 8284 adult participants who reported to receive at least three doses of hepatitis B vaccine. Among them, participants seropositive for hepatitis B surface antigen, indicative of being a chronic hepatitis B carrier, were also excluded (n = 116) and also participants seropositive for anti-hepatitis B core antibody, a marker of natural infection with hepatitis B, were excluded (n = 523). This study included 7645 participants; their clinical characteristics are shown in Table 1. The sample weighted prevalence of positive hepatitis B surface antigen (0.42%) and positive anti-hepatitis B core antibody (4.85%) was consistent with the reported prevalence in the US population^9,10^.

**Table 1:** Clinical characteristics of included participants.

	All participants^a^		NGT^b^		AGT^c^		DM^d^		P^e^
n	7645		4639		1989		1017		
Age (year)	39 ± 15		34 ± 13		44 ± 16		53 ± 15		< 0.0001
Gender (female)	4470	58.47%	2896	62.43%	1013	50.93%	561	55.16%	< 0.0001
Body mass index (kg/m^2^)	29.14 ± 7.37		27.46 ± 6.46		30.70 ± 7.54		33.74 ± 8.27		< 0.0001
Hemoglobin A1C (%)	5.6 ± 1.0		5.2 ± 0.3		5.6 ± 0.4		7.1 ± 1.9		< 0.0001
Hemoglobin A1C (mmol/mol)	37 ± 11		33 ± 3		38 ± 4		55 ± 20		< 0.0001
FPG (mg/dL)	104 ± 32		90 ± 6		103 ± 8		148 ± 63		< 0.0001
2hPG (mg/dL)	110 ± 43		92 ± 21		117 ± 32		197 ± 82		< 0.0001
**Racial/ethnic groups**									< 0.0001
Mexican Americans	1053	13.77%	614	13.24%	279	14.03%	160	15.73%	
Other Hispanics	742	9.71%	420	9.05%	209	10.51%	113	11.11%	
Non-Hispanic whites	3225	42.18%	2,122	45.74%	748	37.61%	355	34.91%	
Non-Hispanic blacks	1707	22.33%	913	19.68%	518	26.04%	276	27.14%	
Other	918	12.01%	570	12.29%	235	11.81%	113	11.11%	

Unweighted mean ± standard deviation or n with percent.

*NGT* normal glucose tolerance, *AGT* abnormal glucose tolerance, *DM* diabetes mellitus, *FPG* fasting plasma glucose, *2hPG* 2-h post-challenged plasma glucose.

^a^For all participants: n = 7637 for A1C; n = 3748 for FPG; and n = 2788 for 2hPG.

^b^For NGT: n = 4636 for A1C; n = 1836 for FPG; and n = 1433 for 2hPG.

^c^For AGT: n = 1986 for A1C; n = 1343 for FPG; and n = 1159 for 2hPG.

^d^For DM: n = 1015 for A1C; n = 569 for FPG; and n = 196 for 2hPG.

^e^ANOVA for the three states of glucose tolerance.

### The impact of glucose tolerance state on seropositivity rate after hepatitis B vaccination

We compared hepatitis B seropositivity among vaccinated NGT, AGT, and DM groups (as defined in “Methods”). Sample-weighted analyses affected and increased seropositivity rates by 2–5% (Table 2). We observed a progressive decline of the seropositivity rate from the NGT group (53.64%) to the AGT group (45.52%) to the DM group (28.84%, P < 0.0001). Although the numeric changes were less impressive (8.12% in the AGT group and 24.80% in the DM group from the NGT group), this reflected a drastically proportional reduction: 15.15% reduction of seropositivity in the AGT group and a 46.25% reduction of seropositivity in the DM group when compared to that in the NGT group.

**Table 2:** The impact of glucose tolerance on seropositivity rates (%, with 95% confidence intervals [CI]) after hepatitis B vaccination.

	Total	Unweighted	Weighted^a^
	n	n, (%)	%, (95%CI)
Normal glucose tolerance (NGT)	4639	2340	53.64%
		(50.44%)	(53.63–53.66)
Abnormal glucose tolerance (AGT)	1989	810	45.52%
		(40.72%)	(45.49–45.54)
Diabetes mellitus (DM)	1017	266	28.84%
		(26.16%)	(28.80–28.87)

^a^P < 0.0001 for weighted percent among three states of glucose tolerance.

### The influence of gender on seropositivity rate after hepatitis B vaccination

Men had a lower seropositivity rate after hepatitis B vaccination compared to that of women in all three states of glucose tolerance (P < 0.0001, Table 3), but within each gender, we again observed a stepwise decrease in seropositivity rate from the NGT to the AGT to the DM group (P < 0.0001 for both genders). In men, seropositivity was reduced in the AGT and DM groups by 16.04% and 51.93% in proportion, respectively, compared to the NGT group. In women, seropositivity was reduced in the AGT and DM groups by 12.17% and 41.30% in proportion, respectively, compared to the NGT group. A gender-standardized analysis confirmed the stepwise change (P < 0.0001).

**Table 3:** The influence of gender on seropositivity rates (%, with 95% confidence intervals [CI]) after hepatitis B vaccination, separated by glucose tolerance state.

	Normal glucose tolerance (NGT)			Abnormal glucose tolerance (AGT)			Diabetes mellitus (DM)		
	Total	Unweighted	Weighted	Total	Unweighted	Weighted	Total	Unweighted	Weighted
	n	n, (%)	%, (95%CI)	n	n, (%)	%, (95%CI)	n	n, %	%, (95%CI)
**A. Gender**^**a**^									
Female	2896	1538	56.39%	1013	455	49.53%	561	160	33.10%
		(53.11%)	(56.37–56.41)		(44.92%)	(49.49–49.56)		(28.52%)	(30.05–31.15)
Male	1743	802	49.30%	976	355	41.39%	456	106	23.70%
		(46.01%)	(49.28–49.33)		(36.37%)	(41.36–41.43)		(23.25%)	(23.65–23.75)
**B. Gender-standardized**^**b**^									
Gender-standardized	4641	2328	53.40%	1987	821	46.11%	1017	267	28.90%
		(50.15%)	(53.38–53.41)		(41.32%)	(46.09–46.14)		(26.30%)	(28.87–28.94)

^a^P < 0.0001 for the weighted percent between both genders in all three states of glucose tolerance states and for weighted percent among three states of glucose tolerance in both genders.

^b^P < 0.0001 for weighted percent among three states of glucose tolerance.

### The influence of race/ethnicity on seropositivity rate after hepatitis B vaccination

Mexican Americans had the lowest seropositivity rate, followed in order of increasing rate by other Hispanic, non-Hispanic Black, non-Hispanic White, and other race; this trend was observed for all three glucose tolerance states (P < 0.0001, Table 4). We again observed a stepwise decrease in seropositivity rate from the NGT to the AGT to the DM group within all racial/ethnic groups (P < 0.0001). This observation was confirmed using a race/ethnicity-standardized analysis (P < 0.0001).

**Table 4:** The influence of race/ethnicity on seropositivity rates (%, with 95% confidence intervals [CI]) after hepatitis B vaccination, separated by glucose tolerance state.

	Normal glucose tolerance (NGT)			Abnormal glucose tolerance (AGT)			Diabetes mellitus (DM)		
	Total	Unweighted	Weighted	Total	Unweighted	Weighted	Total	Unweighted	Weighted
	n	n, (%)	%, (95%CI)	n	n, (%)	%, (95%CI)	n	n, (%)	%, (95%CI)
**A. Race/ethnicity**^**a**^									
Mexican American	614	233	39.50%	279	81	31.01%	160	29	20.06%
		(37.95%)	(39.45–39.56)		(29.03%)	(30.93–31.09)		(18.13%)	(19.95–20.18)
Other Hispanic	420	175	43.66%	209	61	31.97%	113	25	23.62%
		(41.67%)	(43.59–43.73)		(29.19%)	(31.87–32.07)		(22.12%)	(23.48–23.76)
Non-Hispanic White	2122	1136	55.70%	748	342	48.86%	355	94	29.33%
		(53.53%)	(55.68–55.71)		(45.72%)	(48.83–48.90)		(26.48%)	(29.28–29.37)
Non-Hispanic Black	913	434	48.36%	518	206	41.82%	276	70	26.35%
		(47.54%)	(48.31–48.41)		(39.77%)	(41.75–41.89)		(25.36%)	(26.27–26.44)
Other race	570	362	63.06%	235	120	50.46%	113	48	42.26%
		(63.51%)	(63.00–63.11)		(51.06%)	(50.37–50.55)		(42.48%)	(42.12–42.40)
**B. Race/ethnicity-standardized**^**b**^									
Race/Ethnicity-standardized	4641	2330	53.53%	1,989	816	45.77%	1,014	270	28.96%
		(50.19%)	(53.52–53.55)		(41.04%)	(45.74–45.79)		(26.62%)	(28.92–28.99)

^a^P < 0.0001 for the weighted percent among five racial/ethnic groups in all three glucose tolerance states and for weighted percent among three states of glucose tolerance in five racial/ethnic groups.

^b^P < 0.0001 for weighted percent among three states of glucose tolerance.

### The influence of BMI on seropositivity rate after hepatitis B vaccination

To simplify BMI strata, we used the nearest integer greater than or equal to the BMI value to standardize the influence of BMI on seropositivity rate. We observed a stepwise decline in seropositivity rate from the NGT to the AGT to the DM group in all participants (P < 0.0001, Table 5A). The results were confirmed in subgroup analyses by gender and race/ethnicity (P < 0.0001, Table 5B,C).

**Table 5:** Body mass index-standardized seropositivity rates (%, with 95% confidence intervals [CI]) after hepatitis B vaccination, separated by glucose tolerance state.

	Normal glucose tolerance (NGT)			Abnormal glucose tolerance (AGT)			Diabetes mellitus (DM)		
	Total	Unweighted	Weighted	Total	Unweighted	Weighted	Total	Unweighted	Weighted
	n	n (%)	% (95%CI)	n	n (%)	% (95%CI)	n	n (%)	% (95%CI)
**A. All participants**^**a**^									
	4661	2274	52.07%	1992	846	47.68%	992	296	32.99%
		(48.79%)	(52.05–52.08)		(42.48%)	(47.65–47.60)		(29.80%)	(32.95–33.03)
**B. Gender**^**b**^									
Female	2907	1487	54.67%	1020	483	52.24%	542	183	38.58%
		(51.13%)	(54.65–54.69)		(47.36%)	(52.21–52.28)		(33.78%)	(38.53–38.64)
Male	1759	790	47.99%	967	359	42.85%	449	114	26.23%
		(44.91%)	(47.96–48.01)		(37.11%)	(42.81–42.89)		(25.41%)	(26.18–26.28)
**C. Race/ethnicity**^**c**^									
Mexican American	619	232	38.88%	276	81	31.80%	113	27	21.50%
		(37.41%)	(38.82–38.94)		(29.20%)	(31.72–31.89)		(24.10%)	(21.38–21.62)
Other Hispanic	423	169	42.89%	207	65	33.02%	113	25	25.43%
		(39.93%)	(42.83–42.96)		(31.50%)	(32.92–33.12)		(22.12%)	(25.29–25.57)
Non-Hispanic White	2131	1107	54.02%	750	361	51.45%	345	105	33.82%
		(51.93%)	(54.00–54.04)		(48.09%)	(51.42–51.48)		(30.46%)	(33.78–33.87)
Non-Hispanic Black	916	426	47.35%	520	207	42.16%	271	76	29.37%
		(46.54%)	(47.30–47.40)		(39.86%)	(42.09–42.23)		(28.19%)	(29.27–29.46)
Other	572	355	61.83%	236	124	51.63%	110	51	46.33%
		(62.02%)	(61.78–61.89)		(52.56%)	(51.54–51.73)		(46.56%)	(46.18–46.47)

^a^P < 0.0001 for the weighted percent among three glucose tolerance states.

^b^P < 0.0001 for weighted percent between both genders in all three states of glucose tolerance and for weighted percent among three states of glucose tolerance in both genders.

^c^P < 0.0001 for weighted percent between among 5 racial/ethnic groups in all three states of glucose tolerance and for weighted percent among all three states of glucose tolerance in all 5 racial/ethnic groups.

### The influence of age on seropositivity rate after hepatitis B vaccination

We observed a progressive decline in age-standardized seropositivity rate from the NGT to the AGT to the DM group in all participants (P < 0.0001, Table 6A). Subgroup analyses based on gender and race/ethnicity confirmed a stepwise decline (P < 0.0001, Table 6B,C), except for non-Hispanic Blacks, who showed a significant difference among three states of glucose tolerance (P < 0.0001), mainly due to a significant decrease in the DM group while similar seropositivity rates between the NGT and AGT groups.

**Table 6:** Age-standardized seropositivity rates (%, with 95% confidence intervals [CI]) after hepatitis B vaccination, separated by glucose tolerance state.

	Normal glucose tolerance (NGT)			Abnormal glucose tolerance (AGT)			Diabetes mellitus (DM)		
	Total	Unweighted	Weighted	Total	Unweighted	Weighted	Total	Unweighted	Weighted
	n	n (%)	% (95%CI)	n	n (%)	% (95%CI)	n	n (%)	% (95%CI)
**A. All participants**^**a**^									
	4668	2211	51.65%	1988	874	48.00%	989	331	34.84%
		(47.38%)	(51.63–51.67)		(43.94%)	(47.97–48.02)		(33.46%)	(34.80–34.88)
**B. Gender**^**b**^									
Female	2915	1470	54.57%	1013	493	52.61%	542	190	38.53%
		(50.41%)	(54.55–54.59)		(48.69%)	(52.57–52.65)		(35.10%)	(38.48–38.58)
Male	1758	747	46.96%	967	377	43.64%	450	138	30.10%
		(42.50%)	(46.93–46.98)		(39.03%)	(43.60–43.67)		(30.73%)	(30.04–30.15)
**C. Race/ethnicity**^**c**^									
Mexican American	629	223	37.49%	277	88	33.88%	147	32	23.65%
		(35.49%)	(37.43–37.54)		(31.66%)	(33.79–33.97)		(21.82%)	(23.52–23.77)
Other Hispanic	427	163	41.42%	210	67	33.89%	105	30	31.80%
		(38.17%)	(41.35–41.48)		(32.16%)	(33.79–33.99)		(29.01%)	(31.64–31.96)
Non-Hispanic White	2140	1088	53.65%	744	370	51.89%	341	114	35.42%
		(50.86%)	(53.63–53.67)		(49.72%)	(51.86–51.92)		(33.32%)	(35.37–35.47)
Non-Hispanic Black	915	396	44.79%	521	225	45.19%	271	89	33.35%
		(43.25%)	(44.74–44.83)		(43.24%)	(45.12–45.26)		(32.84%)	(33.25–33.44)
Other race	581	355	61.03%	228	122	52.55%	109	53	48.38%
		(61.11%)	(60.98–61.09)		(53.55%)	(52.45–52.64)		(48.47%)	(48.23–48.52)
**D. Body mass index**^**d**^									
BMI < 25.0	1896	1035	58.27%	429	227	54.94%	113	48	45.51%
		(54.60%)	(58.25–58.30)		(53.02%)	(54.88–54.99)		(42.39%)	(45.39–45.64)
BMI 25.0–29.9	1452	674	51.02%	635	274	47.26%	245	86	36.38%
		(46.43%)	(51.00–51.05)		(43.10%)	(47.22–47.31)		(35.20%)	(36.31–36.46)
BMI ≥ 30.0	1321	534	44.04%	907	343	43.30%	648	195	31.37%
		(40.43%)	(44.01–44.07)		(37.82%)	(43.26–43.34)		(30.07%)	(31.33–31.42)

^a^P < 0.0001 for the weighted percent among three glucose tolerance states.

^b^P < 0.0001 for weighted percent between both genders in all three states of glucose tolerance and for weighted percent among three states of glucose tolerance in both genders.

^c^P < 0.0001 for weighted percent between among 5 racial/ethnic groups in all three states of glucose tolerance and for weighted percent among all three states of glucose tolerance in all 5 racial/ethnic groups.

^d^P < 0.0001 for weighted percent between among 3 BMI categories in all three states of glucose tolerance and for weighted percent among all three states of glucose tolerance in all 3 BMI categories.

We were not able to perform the analysis standardized by both age and BMI, due to limited sample size and complexity of analyses. Alternatively, we stratified the BMI into 3 categories based the widely accepted criteria. We observed similar trends in age-standardized seropositivity rate among three states of glucose tolerance within subgroups stratified by BMI (P < 0.0001, Table 6D), although the difference between NGT and AGT groups was less drastic in obese participants (BMI ≥ 30). Again, a progressive decline in seropositivity rate among three BMI categories was noted in all states of glucose tolerance (P < 0.0001).

## Discussion

In this cohort, we observed that a progressive decrease in seropositivity rate after hepatitis B vaccination was associated with deterioration of glucose tolerance. We confirmed this association in subgroup analyses stratified by gender and race/ethnicity and in BMI-standardized analyses. Age-standardized analyses confirmed a stepwise decline in seropositivity rate as glucose tolerance deteriorated in all groups except non-Hispanic Blacks, mainly with similar seropositivity rates between the NGT and AGT groups while a significant decrease in the DM group. Regardless of this exception, we observed a significant decrease in seropositivity rate in the DM group compared to the NGT and AGT groups throughout all our analyses.

Vaccination is an important and highly effective measure in the prevention of certain infectious diseases, including hepatitis B. The CDC began recommending hepatitis B vaccination for all newborns in the United States in 1991 after the first hepatitis B vaccine was approved by the FDA in1981. A study conducted before hepatitis B vaccination was widely available/recommended revealed that the prevalence of hepatitis B surface antibody was twice as high in people with DM as in controls^11^. The CDC also reported that the rate of acute hepatitis B infection was approximately twice as high among adults with DM compared to adults without DM^12^. DM was independently associated with an increased risk for acute hepatitis B among adults without hepatitis B risk behaviors^13^. Furthermore, the association of hepatitis B outbreak with reusable lancet devices for blood glucose monitoring was well-recognized^14^. Although hepatitis B vaccination is strongly recommended for patients with DM, there is a paucity of information regarding the immunogenicity of the hepatitis B vaccine in this group. A small study of 71 patients with DM reported a > 90% seropositive response at month 13^15^. Although a systematic review of the literature reported similar immunogenicity in people with and without DM^16^, a lower seropositivity rate was reported in those with DM (75.0% vs. 96.9% in controls, P = 0.01)^17^, which was also recently observed in a phase 3 clinical trial of a hepatitis B vaccine^18^. Thus, a lower seropositive response to hepatitis B vaccination in patients with DM is not totally unexpected, even though its clinical significance has not been addressed before. In line with previous findings, we observed a stepwise reduction in seropositivity in response to hepatitis B vaccination, from individuals with NGT to those with AGT, and then to those with DM, a distinction that has not been reported before.

It is well-recognized that obese individuals are more likely to have a lower seropositivity rate in response to hepatitis B vaccination than non-obese individuals^18–21^. Obesity is a well-recognized risk factor for type 2 diabetes; consistent with this, we observed a stepwise increase in BMI in individuals with NGT to those with AGT to those with DM (P < 0.0001, Table 1). Upon BMI standardization, we observed a stepwise reduction in seropositivity rate in all participants with deterioration of glucose tolerance, as well as in subgroup analyses (Table 5). These observations confirm that the impact of glucose tolerance state on seropositive response to hepatitis B vaccination is independent of the effect of BMI.

A reduced seropositive response to hepatitis B vaccination is also associated with aging^18^. To dissect the interaction of age with seropositive response to hepatitis B in this cohort, we compared seropositivity rates using age standardization, which confirmed a progressive decrease of seropositivity rate with decline of glucose tolerance (Table 6A). Subgroup analyses confirmed a stepwise decline in age-standardized seropositivity rates as glucose tolerance deteriorated (Tables 6B–D), except for similar seropositivity rates between the NGT and AGT groups in non-Hispanic Blacks. Nevertheless, we observed a significantly lower seropositivity rate in the DM group when compared to the NGT and AGT groups in all analyses. These observations confirm the independent impact of glucose tolerance state on seropositive response to hepatitis B vaccination.

Persistence of anti-hepatitis B sureface antibody was observed after hepatitis B vaccination^22^. In contrast to the seropositive response (> 90%) observed in most hepatitis B vaccination trials which assessed response within 12 months after vaccination, the greatest seropositive response we observed was only 61.83%, in the NGT participants from other racial/ethnic group. The serological titer of antibodies declines with time after the initial vaccination. The lower seropositivity rates that we observed in the present cohort could be due to a lapse of time from vaccination to the measurement of serological markers. However, the lowest seropositivity rate we observed in the DM group cannot be explained by lapse of time alone, as a previous clinical trial noted a lower seropositivity rate in participants with DM (58.3% vs. 78.1% for Engerix-B and 83.3% vs. 92.0% for Sci-B-Vac, diabetic vs. non-diabetic, respectively) at 4 weeks after the final dose^18^. Furthermore, the seropositivity rates observed shortly after vaccination in patients with DM in that study are still much higher than rate that we observed (28.84%) in the DM group in our cohort. Thus, the exceptionally low seropositivity rate we observed in the DM group is most likely a combination of initial reduced seropositivity immediately after hepatitis B vaccination and diminished seropositivity with the lapse of time. The question that remains to be addressed is whether there is any difference in the rate of decline in serological titers by glucose tolerance state, which cannot be properly answered from this cross-sectional cohort. A longitudinal study to compare rates of decline among participants with varying glucose tolerance states is required to answer this question.

The most intriguing observation of the present study is the stepwise reduction in seropositivity rates from the NGT to the AGT group, and then to the DM group, which cannot be explained by the impact of BMI or age as demonstrated in our BMI—(Table 5) and age—(Table 6) standardized analyses. This has not been reported in the literature before. Multiple abnormalities in cell-mediated immunity have been noted in patients with DM^23^. Diabetes is associated with a chronic inflammatory state^24^, which provides a link between metabolic dysregulation and immunological imbalance^25^. Furthermore, altered immune and inflammatory responses are also observed in prediabetic individuals, with a progression in immune and inflammatory biomarker profiles upon the development and progression of type 2 diabetes^26^. Thus, AGT before the onset of overt DM could also have an impact on immunogenicity, leading to a lower seropositive response to hepatitis B vaccination than that in individuals with NGT, but not as low as in those with DM.

With standardization and subgroup analyses, the difference in seropositivity rate decreased in the AGT and DM groups when compared to the NGT group. Given that metabolic derangement is less pronounced in the AGT group compared to that in the DM group, the difference in seropositivity rate in the AGT vs. NGT group diminished in the age-standardized analysis in non-Hispanic Blacks (Table 6C), whereas the seropositivity rate in the AGT group remained significantly lower than that of the NGT group for all other analyses, even in age-standardized analysis of obese participants with BMI ≥ 30.0 (43.30% in the AGT group and 44.04% in the NGT group, P < 0.0001). Nevertheless, seropositivity rate in the DM group was significantly lower than in the AGT and NGT groups for all comparisons (P < 0.0001 for all). Seropositivity was as low as 20.06% in Mexican Americans (Table 4A), which raises a significant concern about the effectiveness and efficacy of hepatitis B vaccination in patients with DM in this demographic. Thus, regular follow-up screening for hepatitis B surface antibody after initial hepatitis B vaccination should be recommended for patients with DM, with an additional booster if indicated^27,28^. A central issue is whether people with DM have a lower seropositivity rate to other vaccines or not, especially COVID-19. A recent study demonstrated a similar humoral response after COVID-19 infection in both diabetic and non-diabetic individuals^29^. However, a seropositivity rate for the COVID-19 vaccine in people with DM has not been reported and remains to be investigated; this is especially important during the COVID-19 pandemic, in which patients with DM have a higher mortality rate from COVID-19 infection than non-diabetic patients^30^.

The present study possesses several unique features. To our knowledge, this is the largest sample that has been used to investigate the effect of glucose tolerance state on seropositive response to hepatitis B vaccination. Furthermore, use of sample-weighted analyses allows our observations to be applied to the US population. By using available information for multiple hepatitis B serological markers, we were able to unequivocally assign seropositivity to participants who were positive for hepatitis B surface antibody from hepatitis B vaccination by excluding natural hepatitis B infection (positive anti-hepatitis B core antibody) and chronic hepatitis B carriers (positive hepatitis B surface antigen). Our age- and BMI-standardized analyses confirmed the independent impact of glucose tolerance state on seropositive response rates after hepatitis B vaccination. Again, to our knowledge, this is the very first report of a stepwise reduction in seropositive response to hepatitis B vaccine in individuals with NGT to those with AGT, and then to those with DM.

Due to the cross-sectional nature of the present study and a lack of information regarding date(s) of hepatitis B vaccine administration, we are not able to address the question of time-related decline of anti-hepatitis B surface antibody, which could contribute to the low seropositivity rates observed in participants with AGT and DM. Furthermore, there was not information available about which hepatitis B vaccines the participants received, which could also potentially affect seropositivity rate. However, given that lower seropositivity in people with DM is well recognized^18^, the observed low seropositivity rates could be a combination of both low initial seropositive response after hepatitis B vaccination and the time-related decline of anti-hepatitis B surface antibody. Because the time from vaccination to the measurement of serological markers is not available in this cohort, the seropositive rate may be underestimated. Since the time of vaccination is not available, it is not clear whether the patient received vaccination before or after the development of AGT or DM. Whether the time-related decline rates differ among various glucose tolerance states is beyond the scope of this study and will require a longitudinal study to address this issue. Due to limited sample size, especially that of the DM group, we are not able to perform a crossed standardized analysis by considering all covariates, including gender, race/ethnicity, BMI, and age. However, subgroup analyses of and BMI- and age-standardized seropositivity rates confirmed our observation of a stepwise effect of glucose tolerance state on hepatitis B seropositivity. Although there is no information about the type of diabetes experienced by participants in this cohort, given that most of diabetes is type 2 diabetes, we infer that the observed results are mainly from patients with type 2 diabetes. Because a lower seropositive response has been also reported in patients with type 1 diabetes^17^, it is highly possible that the results observed in this study could also be applied to patients with type 1 diabetes. Of note, the host genetic variants of human leukocyte antigen (HLA) loci are associated with altered response to hepatitis B vaccination^31^.

In summary, we observed a significant decline in hepatitis B seropositivity rate in a stepwise fashion in participants with NGT to those with AGT, and then to those with DM. Due to a fairly low seropositivity rate in people with DM, our observations suggest that a follow-up measurement of hepatitis B surface antibody is required, with additional booster vaccination as indicated, to ensure the risk of hepatitis B infection is reduced in diabetic patients. However, based on the cross-sectional nature of the present study, an interval of reassessment cannot be provided. Whether the decreased seropositivity rate we observed as associated with deterioration of glucose tolerance is unique for hepatitis B, or is a general phenomenon for all vaccination in people with DM, remains to be proven and is especially important during the COVID-19 pandemic.
